# Association of Serum Uric Acid with 2-Hour Postload Glucose in Chinese with Impaired Fasting Plasma Glucose and/or HbA1c

**DOI:** 10.1371/journal.pone.0067759

**Published:** 2013-07-03

**Authors:** Hong-Qi Fan, Wei Tang, Zhi-Xiao Wang, Su-Juan Wang, Yue-Hua Qin, Qi Fu, Yuan Gao, Min Sun, Mei Zhang, Hong-Wen Zhou, Tao Yang

**Affiliations:** 1 Department of Endocrinology, The First Affiliated Hospital of Nanjing Medical University, Nanjing, Jiangsu, China; 2 Department of Endocrinology, The Affiliated Jiangyin Hospital of Southeast University Medical College, Jiangyin, Jiangsu, China; Brigham & Women's Hospital, and Harvard Medical School, United States of America

## Abstract

**Objective:**

To examine whether serum uric acid (SUA) is associated with 2-hour postload glucose (2-h PG) in Chinese with impaired fasting plasma glucose (IFG) and/or HbA1c (IA1C).

**Research Design and Methods:**

Anthropometric and biochemical examinations, such as SUA concentration, were performed in 3763 individuals from all the villages in Baqiao County, China. A 75-g oral glucose tolerance test (OGTT) was conducted in 1197 Chinese with prediabetes as having IFG (110≤ fasting plasma glucose [FPG] <126 mg/dl and HbA1c <6.5%), IA1C (5.7% ≤ HbA1c <6.5% and FPG <126 mg/dl), or both.

**Results:**

The present study included 1197 participants with IFG and/or IA1C (mean age 56.5±10.3 years; 50.6% men). In multivariate linear regression, after adjustment for gender, age, smoking and drinking, body mass index (BMI), systolic and diastolic blood pressure (SBP, DBP), lipid profiles, logarithmic transformed C-reactive protein (log-CRP), estimated glomerular filtration rate (e-GFR), FPG and HbA1c, with a 1-mg/dl increment of SUA, 2-h PG increased by 5.04±0.72 (*P*<0.001), 3.06±1.08 (*P* = 0.001), 5.40±1.26 (*P*<0.001), and 2.34±2.16 mg/dl (*P* = 0.056) in all participants, in participants with normal glucose tolerance (NGT), with impaired glucose tolerance (IGT), and with 2-h newly diagnosed diabetes (2-h NDM, with 2-h PG ≥200 mg/dl), respectively. In both men and women, 2-h PG increased progressively and significantly from the lower to the upper SUA tertiles (*P*<0.001). Moreover, in multivariate logistic regression, 1-standard deviation (SD; 1.53 mg/dl) increment of SUA was significantly associated with a 36% higher risk for 2-h NDM (Odds ratio [CI 95%]: 1.36 [1.09–1.99]; *P = *0.03).

**Conclusions:**

SUA is significantly associated with 2-h PG in Chinese with IFG and/or IA1C.

## Introduction

Serum uric acid (SUA), the end product of purine metabolism, possesses both antioxidant and pro-oxidant properties, which depend on its chemical microenvironment. In clinical investigations, SUA was reported to be associated with gout, hypertension, atherosclerosis, metabolic syndrome, diabetes and prediabetes [1]–[3]. More recently, hyperuricemia was documented in subjects with cardiovascular diseases [4] and recognized as an independent predictor of myocardial infarction and stroke [5].

On the other hand, type 2 diabetes is a recognized and independent risk factor for cardiovascular disease [6]–[8], even in the absence of coronary artery disease or hypertension [9]. Patients with prediabetes (based on impaired fasting glucose [IFG], impaired glucose tolerance [IGT], or impaired HbA1c [IA1C] of 5.7–6.4%) are at high risk of future type 2 diabetes, with 70% of them developing type 2 diabetes within 10 years [10]. More importantly, patients with prediabetes seem to share the similar concomitant damage to end target organs, as patients with diabetes [11].

High blood glucose concentration or elevated HbA1c was shown to be a risk factor for cardiovascular complications, even in nondiabetic individuals [12]. Although the underlying mechanism is still controversial, fasting plasma glucose (FPG) and HbA1c, the most common glycemic indexes, could not completely explain the observed risk. Recently, in the Diabetes Epidemiology: Collaborative Analysis of Diagnostic Criteria in Europe (DECODE) study, it was demonstrated that FPG concentrations alone could not identify individuals at increased risk of cardiovascular complications associated with hyperglycemia, and the oral glucose tolerance test (OGTT) could provide additional prognostic information [13]. In addition, in the Diabetes Control and Complications Trial, it was reported that the degree of glucose load, which was not completely reflected by mean HbA1c, was more strongly associated with the observed risk of cardiovascular diseases [14]. Although many studies have indicated a critical role of postload glucose in the development of complications, the postchallenge values are still frequently being neglected [15]. This is probably due to the inconvenience and costs of an OGTT measurement. Considering the importance of postload hyperglycemia in the DECODE study [13] and others [14], [16], [17], and the crucial role of SUA in the development of cardiovascular diseases [2]–[5], we therefore aimed to investigate the association of SUA and 2-h PG in patients with IFG and/or IA1C.

## Methods

### Study Population

The present study was conducted from January to May in 2010 in the framework of routine health examinations in all the villages in Baqiao County in Gaoyou, a newly established rural residential area about 299 kilometers northwest to Shanghai, China. Study protocol was approved by the Ethics Committee of the First Affiliated Hospital of Nanjing Medical University. From the local authorities, we obtained the population data of 10008 inhabitants aged from 18 to 74 years. Proportionately stratified random sampling was used to select a representative sample from the total population, and the population size was set at 5000 participants. 3918 individuals, out of the 5000 (78.4%), participated after having given informed written consent. 155 individuals were excluded from the present study, because they had established diabetes (n = 82; defined as the use of glucose-lowering agents, or diagnosed diabetes, or both), missing information on history of diabetes (n = 17), FPG (n = 18) or HbA1c (n = 8), self-reported gout or using of allopurinol or uricosuric agents (n = 30). After further exclusion of individuals with normal FPG (<110 mg/dl) and HbA1c (<5.7%) (n = 1831) and newly diabetes (FPG≥126 mg/dl, or HbA1c≥6.5% or both [n = 272]), 1660 individuals, out of the 3763, with IFG (110≤FPG<126 mg/dl and HbA1c<6.5%), or IA1C (HbA1c: 5.7% ≤ HbA1c<6.5% and FPG<126 mg/dl), or both, were selected and invited for further OGTT, and eventually 1197 individuals with prediabetes as having IFG and/or IA1C participated in the present study.

### Field Work

Blood pressure (BP) was measured by trained physician using a mercury sphygmomanometer according to the guidelines of the British Hypertension Society [18]. After at least 5 min rest, BP was measured five times with 2-minute interval on the right arm of each participant in the sitting position. These five readings were averaged for further analysis. Hypertension was defined as systolic BP (SBP) at least 140 mmHg and/or diastolic BP (DBP) at least 90 mmHg, and/or use of antihypertensive medication. The same observer also administered a standardized questionnaire to collect information on medical history, smoking habits, alcohol consumption, and the use of medications. Smoking and drinking were defined as at least smoking one cigarette per day or drinking once per week in the past year, respectively. Body weight and body height were measured in each participant, and BMI was calculated as a ratio of the body weight in kilograms to the square of the height in meters.

### Laboratory Determinations

All laboratory measurements were performed after a fasting of at least 12 h. Plasma glucose (with an intra-assay coefficient of variation [CV] of 2.4% and interassay CV of 3.5%), triglyceride, total and low-density lipoprotein (LDL) and high-density lipoprotein (HDL) cholesterol concentrations, serum creatinine, and SUA were measured by enzymatic methods (Chemistry Analyzer Au2700, Olympus Medical Engineering Company, Japan). Hyperuricemia was defined as ≥7 mg/dl (in men) or ≥6 mg/dl (in women). Serum insulin was assessed in duplicate by a highly specific radioimmunoassay using two monoclonal antibodies (intra-assay CV, 2.5%; interassay CV, 3.7%). C-reactive protein (CRP) was measured by a high-sensitivity turbidimetric immunoassay (Behring, Marburg, Germany). Values of estimated glomerular filtration rate (e-GFR; mL/min/1.73 m^2^) were calculated by using the equation proposed by investigators in the Chronic Kidney Disease Epidemiology (CKD-EPI) Collaboration [19].

A simplified 75-g OGTT was performed with 0-, 30-, and 120-min sampling for plasma glucose and insulin after a fasting of at least 12 h. Then, 1197 participants were classified into three groups according to the OGTT results (normal glucose tolerance [NGT], 2-h PG <140 mg/dl; impaired glucose tolerance [IGT], 140≤2-h PG <200 mg/dl; 2-h newly diabetes mellitus [2-h NDM], 2-h PG ≥200 mg/dl). Hepatic insulin sensitivity was evaluated by HOMA with the equation: (fasting glucose [mmol/L] ×fasting insulin [mU/L])/22.5. Whole body Insulin sensitivity index was evaluated by the Matsuda index with the equation: 10,000/square root of (fasting glucose×fasting insulin) × (mean glucose×mean insulin during OGTT). The Matsuda index is strongly related to the euglycemic hyperinsulinemic clamp, the gold standard assessment of insulin sensitivity [20].

### Statistics Analysis

SAS software, version 9.1 (SAS Institute Inc), was applied for database management and statistical analyses. Skewed variables were log-transformed. Means and proportions were compared by ANOVA and Fisher’s exact test, respectively. Multivariate linear regression was applied to investigate the determinants of 2-h PG in patients with different glucose tolerance (NGT, IGT, 2-h NDM) in a full adjustment model including covariables of age, BMI, current smoking and drinking habits, SBP and DBP, lipid parameters, e-GFR, FPG, HbA1c and logarithmic transformed CRP (log-CRP). Multivariate logistic regression was used to study risk factors of 2-h NDM (0, 1) in a stepwise model, with age, FPG and HbA1C forced in the model. We stratified the participants into three groups by the tertiles of their SUA in men and women, respectively, and investigated their association with 2-h PG by general linear model analysis. Gender interaction analysis was performed by general linear model analysis with gender, SUA and gender*SUA put in the model. Correlations of log-CRP and insulin sensitivity with SUA were studied with Pearson’s method. *P*<0.05 was considered statistically significant.

## Results

### Study Population

Characteristics of the 1197 patients with IFG and/or IA1C are presented in Table 1 according to their glucose tolerance, with NGT (n = 837), IGT (n = 309), and 2-h NDM (n = 51). There was no significant difference among the three groups in drinking (*P = *0.10), DBP (*P* = 0.05), HDL (*P* = 0.05), LDL (*P = *0.05), creatinine (*P* = 0.19), and e-GFR (*P* = 0.52), but a significant difference in gender distribution (*P = *0.01). Furthermore, from NGT to 2-h NDM group, a gradual and significant increasing trend was observed in age (*P = *0.001), BMI (*P*<0.001), SBP (*P*<0.001), total cholesterol (*P* = 0.04), triglycerides (*P*<0.001), Log-CRP (*P* = 0.007), FPG (*P*<0.001), HbA1c (*P*<0.001), SUA (*P = *0.002) and proportion of hyperuricemia (*P = *0.002) and hypertension (*P*<0.001), but a decreasing trend in the proportion of smoking (*P = *0.03).

**Table 1:** Characteristics in patients with prediabetes according to glucose tolerance.

Variables	All (n = 1197)	NGT (n = 837)	IGT (n = 309)	2-h NDM (n = 51)	*P*
Male gender, n (%)	606 (50.6)	447 (53.4)	136 (44.0)	23 (45.1)	0.01
Age, years	56.5±10.3	55.8±10.6	57.5±9.4	60.8±8.8	0.001
Smoking (%)	430 (35.9)	321 (38.4)	95 (30.7)	14 (27.5)	0.03
Drinking (%)	285 (23.7)	211 (25.2)	67 (21.7)	7 (13.7)	0.10
BMI, kg/m^2^	24.9±3.2	24.5±3.1	25.5±3.3	25.7±3.5	<0.001
SBP, mm Hg	141.4±20.1	139.8±19.7	145.1±21.1	146.6±20.7	<0.001
DBP, mm Hg	87.6±9.8	87.2±9.8	88.7±9.7	88.2±8.9	0.05
Hypertension (%)	320 (26.7)	189 (22.6)	105 (34.0)	26 (51.0)	<0.001
Total cholesterol, mmol/l	5.14±0.99	5.09±1.00	5.24±0.96	5.31±1.01	0.04
HDL cholesterol, mmol/l	1.30±0.31	1.31±0.30	1.27±0.31	1.26±0.30	0.05
LDL cholesterol, mmol/l	3.06±0.71	3.03±0.72	3.14±0.69	3.15±0.72	0.05
Triglycerides, mmol/l	1.64±1.53	1.51±1.36	1.91±1.88	2.04±1.76	<0.001
Log-CRP, mmol/l	0.92±0.91	0.86±0.95	1.03±0.86	1.19±0.78	0.007
Creatinine, mmol/l	68.1±16.3	68.7±16.4	66.7±16.3	68.9±14.9	0.19
FPG, mg/dl	104.40±12.60	102.62±10.87	108.04±12.72	111.62±10.86	<0.001
2-h PG, mg/dl	7.0±1.3	5.9±1.2	9.0±0.9	12.3±1.2	<0.001
HbA1c, %	5.9±0.3	5.9±0.3	6.0±0.3	6.2±0.4	<0.001
SUA, mg/dl	5.1±1.5	5.0±1.5	5.3±1.6	5.6±1.8	0.001
Hyperuricemia (%)	170 (14.2)	103 (12.3)	53 (17.2)	14 (27.5)	0.002
e-GFR, ml/min per 1.73 m^2^	97.8±16.0	98.4±16.1	98.0±16.2	97.8±14.2	0.52

Data were presented as mean (± SD) or number (%). Means and proportions were compared by ANOVA and Fisher’s exact test, respectively. *P* values testing the overall difference among NGT, IGT and 2-h NDM groups. Definitions of hypertension, hyperuricemia, 2-h NDM (2-h newly diagnosed diabetes) were in methods. BMI, body mass index; SBP, systolic blood pressure; DBP, diastolic blood pressure; HDL, high density lipoprotein; LDL, low density lipoprotein; log-CRP, logarithmic transformed C-reactive protein; FPG, fasting plasma glucose; 2-h PG, 2 hour postload glucose; SUA, serum uric acid; e-GFR, estimated glomerular filtration rate. International system of units (SI) conversion: plasma glucose 1 mg/dl = 1/18 mmol/l; SUA 1 mg/dl = 59.5 µmol/l.

### Multivariate Analyses

Multiple lineal regression analysis was performed to test the independent association between SUA and 2-h PG, with adjustment for gender, age, smoking and drinking, BMI, SBP, DBP, total cholesterol, HDL and LDL, triglycerides, log-CRP, e-GFR, FPG and HbA1c. With a 1-mg/dl increment of SUA, 2-h PG increased by 5.04±0.72 (*P*<0.001), 3.06±1.08 (*P = *0.001), 5.40±1.26 (*P*<0.001), and 2.34±2.16 mg/dl (*P = *0.056) in all participants, in participants with NGT, with IGT, and with 2-h NDM, respectively (Table 2). Other independent predictors of 2-h PG were age, BMI, SBP, FPG and HbA1c in all participants; SBP and FPG in participants with NGT; smoking, LDL and HbA1c in participants with IGT; and smoking in participants with 2-h NDM.

**Table 2:** Muiltiple linear regression analysis between 2-h PG and different covariates in the entire study population and in groups with different glucose tolerance.

	All (n = 1197)		NGT (n = 837)		IGT (n = 309)		2-h NDM (n = 51)	
	β ± SE	*P*	β ± SE	*P*	β ± SE	*P*	β ± SE	*P*
SUA, mg/dl	5.04±0.72	<0.001	3.06±1.08	0.001	5.40±1.26	<0.001	2.34±2.16	0.056
Age, +10 years	0.20±0.06	0.001	0.07±0.04	0.10	−0.01±0.01	0.87	0.19±0.29	0.53
Smoking (1 = yes, 2 = no)	0.21±0.15	0.18	0.10±0.11	0.38	0.31±0.14	0.03	0.82±0.76	0.04
Drinking (1 = yes, 2 = no)	0.09±0.15	0.55	0.07±0.11	0.50	0.03±0.15	0.82	0.46±0.78	0.56
BMI, kg/m^2^	0.05±0.02	0.006	0.02±0.01	0.18	0.02±0.02	0.27	0.02±0.06	0.76
SBP, +10 mm Hg	0.11±0.04	0.06	0.06±0.03	0.05	0.04±0.03	0.17	0.1±0.1	0.41
DBP, +10 mm Hg	0.02±0.08	0.75	0.08±0.06	0.88	0.02±0.07	0.74	0.2±0.3	0.62
Total cholesterol, mmol/l	0.23±0.24	0.33	0.42±0.16	0.80	0.42±0.24	0.09	1.73±0.99	0.09
HDL cholesterol, mmol/l	−0.15±0.32	0.64	−0.31±0.23	0.16	−0.56±0.31	0.07	−1.10±1.22	0.37
LDL cholesterol, mmol/l	0.24±0.29	0.41	0.09±0.20	0.63	0.59±0.30	0.05	1.76±1.27	0.17
Triglycerides, mmol/l	0.10±0.07	0.13	0.01±0.05	0.83	0.09±0.06	0.14	0.14±0.19	0.45
Log-CRP, mmol/l	0.03±0.05	0.59	0.03±0.04	0.43	0.02±0.05	0.75	0.13±0.31	0.67
FPG, mg/dl	0.64±0.08	<0.001	0.22±0.07	0.001	0.13±0.07	0.06	0.43±0.29	0.14
HbA1c, %	1.24±0.21	<0.001	0.08±0.16	0.60	0.49±0.19	0.01	1.10±0.64	0.09
e-GFR, ml/min per 1.73 m^2^	0.002±0.002	0.32	−0.001±0.002	0.39	0.002±0.003	0.49	0.001±0.013	0.93

Multiple linear regression analysis was performed to investigate the determinants of 2-h postload glucose, after adjustment for gender, age, smoking and drinking, BMI, SBP, DBP, total cholesterol, HDL and LDL, triglycerides, log-CRP, e-GFR, FPG and HbA1c. Values were regression coefficient (β) ± standard error (SE). Definitions of NGT (normal glucose tolerance), IGT (impaired glucose tolerance) and 2-h NDM (2-h newly diagnosed diabetes) were in methods. SUA, serum uric acid; BMI, body mass index; SBP, systolic blood pressure; DBP, diastolic blood pressure; HDL, high density lipoprotein; LDL, low density lipoprotein; log-CRP, logarithmic transformed C-reactive protein; FPG, fasting plasma glucose; e-GFR, estimated glomerular filtration rate. International system of units (SI) conversion: plasma glucose 1 mg/dl = 1/18 mmol/l; SUA 1 mg/dl = 59.5 µmol/l.

**Figure 1:**
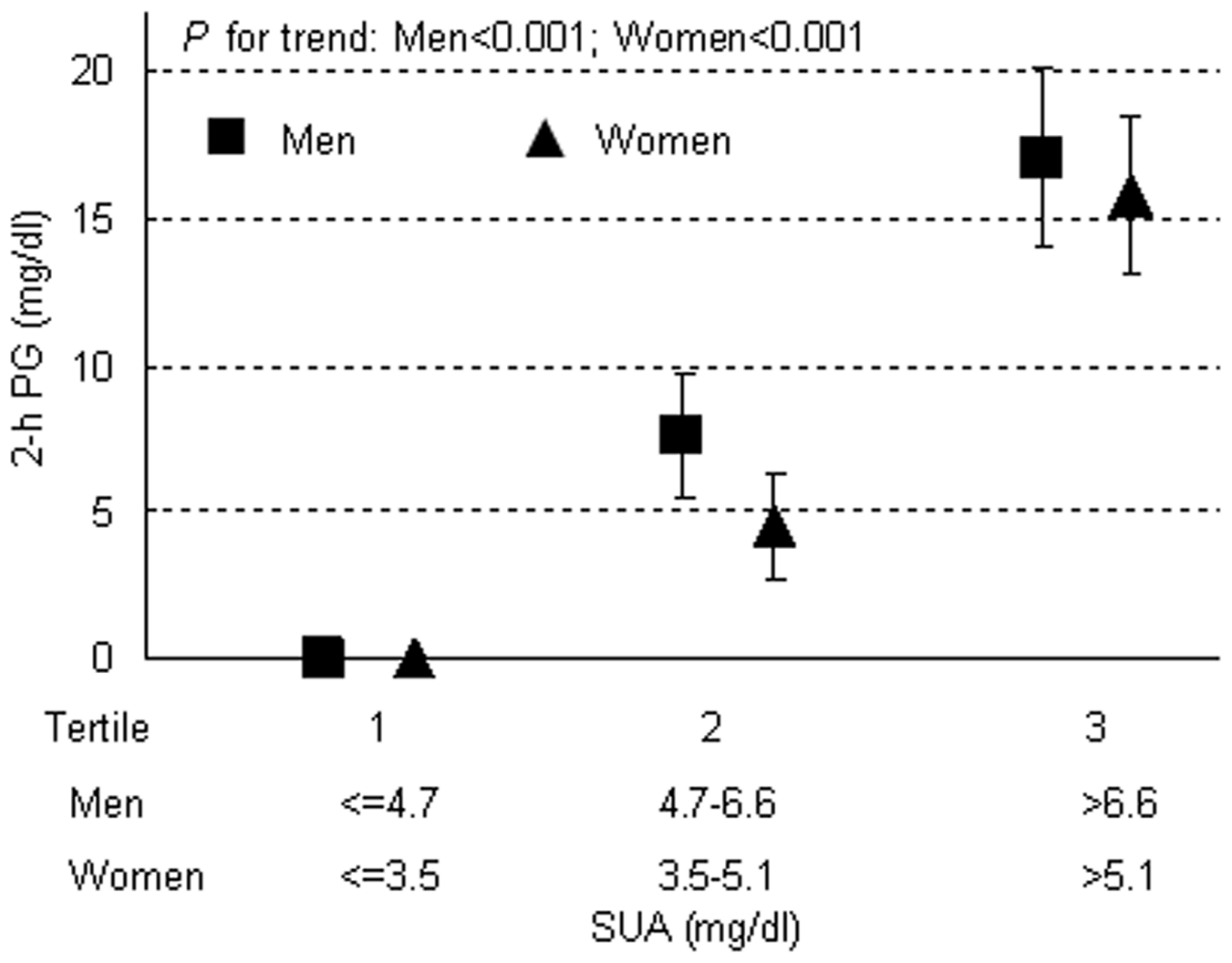
2-h PG levels across the gender-specific tertiles of SUA in men (▪) and women (▴). Mean 2-h PG levels across gender-specific tertiles of SUA resulting from a general linear model were present, with adjustment for age, smoking and drinking, BMI, SBP, DBP, total cholesterol, HDL and LDL, triglycerides, log-CRP, FPG, HbA1c and e-GFR. Vertical lines denoted standard error (SE). Reference values of 2-h PG were111.78 mg/dl in men and 125.10 mg/dl in women. 2-h PG, 2 hour postload glucose; SUA, serum uric acid; BMI, body mass index; SBP, systolic blood pressure; DBP, diastolic blood pressure; HDL, high density lipoprotein; LDL, low density lipoprotein; log-CRP, logarithmic transformed C-reactive protein; FPG, fasting plasma glucose; e-GFR, estimated glomerular filtration rate. International system of units (SI) conversion: plasma glucose 1 mg/dl = 1/18 mmol/l; SUA 1 mg/dl = 59.5 µmol/l.

In Figure 1, 2-h PG levels across gender-specific tertiles of SUA were present. In multivariate general linear regression, 2-h PG gradually and significantly increased from the lower to the upper SUA tertiles in both genders. Although women had a higher 2-h PG than men at each tertile of SUA levels, there was no significant interaction between the gender and the SUA level (*P* for interaction = 0.89).

**Figure 2:**
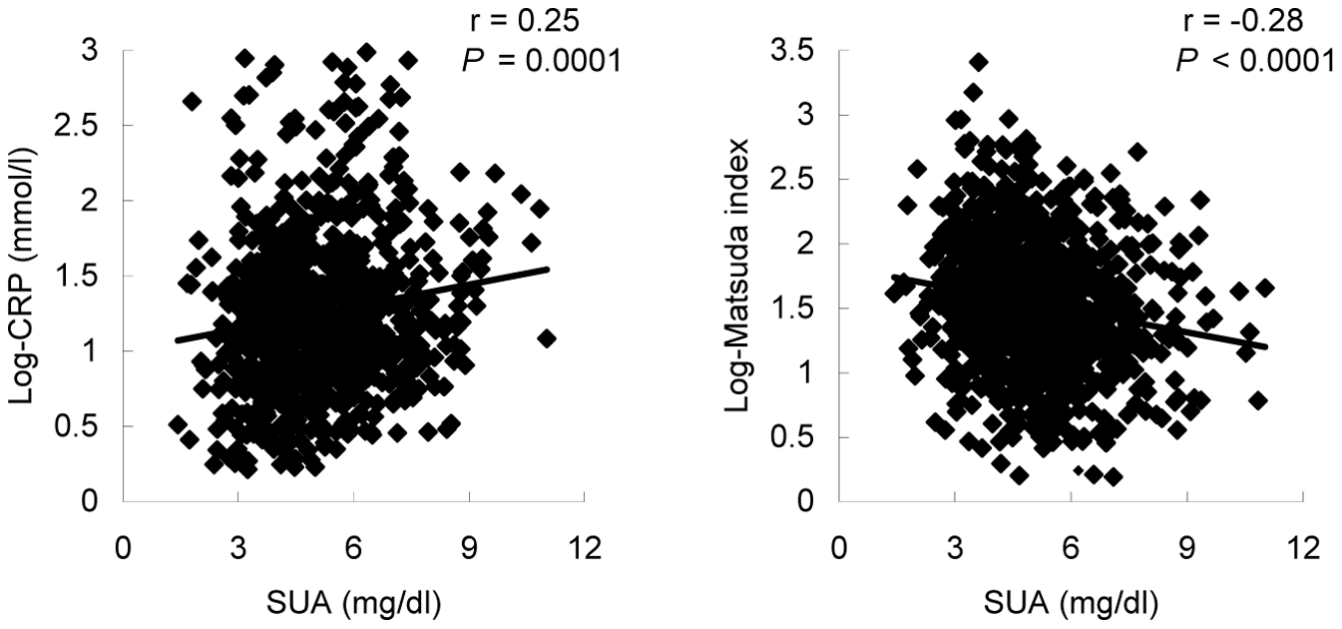
Relation between SUA and log-CRP and insulin sensitivity. Insulin sensitivity was calculated by Matsuda index. SUA, serum uric acid; log-CRP, logarithmic transformed C-reactive protein. International system of units (SI) conversion: plasma glucose 1 mg/dl = 1/18 mmol/l; SUA 1 mg/dl = 59.5 µmol/l.

In Table 3, multivariate stepwise logistic regression was performed to determine the influential factors of 2-h NDM, with adjustment for age, FPG and HbA1C forced in the model and gender, smoking and drinking, BMI, SBP, DBP, total cholesterol, HDL and LDL, triglycerides, SUA, e-GFR and log-CRP. In addition to age, FPG and HbA1c, SUA was the only factor significantly associated with 2-h NDM (OR = 1.36 [1.09–1.99]; *P* = 0.03).

**Table 3:** Multivariate logistic analysis of the risk factors for 2-h NDM (n = 1197).

	OR (95% CI)	*P*
Forced variables		
Age, years	1.63 (1.14–2.30)	0.007
HbA1c, %	1.89 (1.44–2.48)	<0.001
FPG, mg/dl	1.70 (1.28–2.25)	<0.001
**Selected**		
SUA, mg/dl	1.36(1.09–1.99)	0.03

Multivariate stepwise logistic regression was performed to determine the influential factors of 2-h NDM (0, 1), in which age, FPG and HbA1C were forced in the model and gender, smoking and drinking, BMI, SBP, DBP, total cholesterol, HDL and LDL, triglycerides, SUA, log-CRP and e-GFR were considered as potential influential factors. OR (odds ration) and 95% CI (confidence interval) was calculated per 10 years in age and per 1-SD in other quantitative variables and presence against absence in qualitative variables. Only significant influential factors were shown in the table. 2-h NDM, 2-h newly diabetes mellitus; BMI, body mass index; SBP, systolic blood pressure; DBP, diastolic blood pressure; FPG, fasting plasma glucose; HDL, high density lipoprotein; LDL, low density lipoprotein; SUA, serum uric acid; log-CRP, logarithmic transformed C-reactive protein; e-GFR, estimated glomerular filtration rate. International system of units (SI) conversion: plasma glucose 1 mg/dl = 1/18 mmol/l; SUA 1 mg/dl = 59.5 µmol/l.

Of note, SUA was also significantly associated with log-CRP (r = 0.25, *P* = 0.001) and whole body insulin sensitivity calculated by Matsuda index (r = −0.28, *P*<0.001) (Fig. 2) but not hepatic insulin sensitivity calculated by HOMA (r = −0.06, *P* = 0.084).

## Discussion

The main finding of this study was that elevated SUA in individuals with prediabetes, defined as IFG and/or IA1c, was significantly associated with higher 2-h PG and risk of type 2 diabetes, independent of FPG, HbA1c and other recognized risk factors.

Currently, gout and renal disorders were considered as the main consequences of hyperuricemia, and SUA was recognized as a potential risk factor for hypertension [1], [21], stroke [5], [22], and cardiovascular diseases [23] More recently, some studies suggested that type 2 diabetes was strongly associated with hyperuricemia, and lowering SUA was related to the decreased incidence of diabetes [24]. Some epidemiological studies also indicated that mild-to-moderate elevated FPG was positively associated with SUA, whereas modest or extremely elevated FPG was inversely associated with SUA [25]–[27]. To data, there were limited studies on the relation between SUA and postload plasma glucose, particularly in pre-diabetes, and even the available data were controversial [26], [28]. For instance, in the Qingdao study [27], SUA was negatively and significantly associated with 2-h PG at the higher range of the 2-h PG distribution (2-h PG ≥144 mg/dl), but not at the normal range of 2-h PG. However, in another clinical study, SUA was significantly and positively correlated with 2-h PG at the normal range of 2-h PG in nondiabetic Mauritian subjects [28]. Our study, for the first time, demonstrated that SUA in individuals with prediabetes (IFG and/or IA1C) was positively and significantly associated with 2-h PG, independent of FPG, HbA1c and additional recognized risk factors. Furthermore, we also demonstrated that SUA levels had significant impact on the development of diabetes in patients with IFG and/or IA1c, since 1-SD (1.53 mg/dl) increment in SUA signaled a 36% higher risk for type 2 diabetes. Interestingly, a recent study also showed that SUA was strongly associated with 1-h PG in hypertensive NGT individuals, and similar finding could also be observed in hypertensive IGT and diabetes [29]. Our study, together with above mentioned studies, indicated that SUA might be a major determinant of 2-h PG in patients with prediabetes, suggesting the crucial role of SUA in the deterioration of glucose tolerance.

A recent study showed that the prevalence of prediabetes was approximately 15% in China [30] and many of them would eventually develop type 2 diabetes [10], [31]. Moreover, the proportion of undiagnosed diabetes is also high in China, accounting for 50–80% of the diabetic population [32]–[34]. Considering the growing burden of prediabetes in China, it is practically important to find a simple and portable measurement, especially in rural areas, to screen patients (IFG and/or IA1C) at relatively high risk of future type 2 diabetes, in terms of early prevention and intervention. Indeed, about half of undiagnosed diabetes in Asia actually met the criteria of diabetes by their elevated 2-h PG in an OGTT, but with their fasting glucose in normal range [35], and previous studies demonstrated that the risk of future diabetes in prediabetes was, at least partly, dependent on their postload glucose level or glucose tolerance status [36], [37]. Therefore, the current recommendation is to perform an OGTT in individuals with IFG and/or IA1C to better assess their risk of future type 2 diabetes [38]. However, because of some traditional superstition in China, examination with frequent blood sampling, like OGTT, is thought to be harmful to people’s health, so in many areas in China, especially in the rural area, OGTT is not only a time-consuming and expensive test, but also an “invasive” one. For this reason, OGTT as a mass screening tool may not be suitable in low-income rural areas with limited resources for medical care. In our study, 1-SD increment in SUA levels signaled a 36% higher risk for type 2 diabetes. Therefore, if confirmed, this simple and inexpensive measurement of SUA levels in individuals with IFG and/or IA1C would help to screen patients at relatively low risk of future type 2 diabetes, who would not need for further OGTT. This finding would be important, considering the heavy economic burden and expected increasing incidence of type 2 diabetes in rural areas of China. On the other hand, our study also add to the growing body of evidence suggesting that SUA control would be beneficial in the prevention or delay of the deterioration of glucose tolerance in populations at high risk of diabetes.

In consistence with our findings, previous studies also indicated the significant association between SUA and insulin resistance predominantly in skeletal muscles [39], [40], and the elevated postload glucose would be attributed to the skeletal muscles insulin sensitivity [41], the primary contributor to the whole body insulin sensitivity. This association of SUA with skeletal muscles insulin resistance could be explained by the proinflammatory effects of SUA, namely interfering with postload glucose uptake and impairing blood flow to skeletal muscles [39], [40]. In addition, SUA reduces endothelial NO bioavailability in humans [42], animals and cell [43], [44], increases CRP and oxygen free radicals production [44], and exerts a direct scavenging effect [45], [46]. All factors would interfere with skeletal muscles insulin sensitivity and eventually result in the elevated 2-h PG. In keeping with this, the present study and others showed that CRP was significantly related to SUA (Fig. 2) even after adjustment for other potential confounders (data not shown), confirming the role of SUA-induced inflammation in skeletal insulin resistance. Of note, our findings also showed that whole body insulin sensitivity, but not hepatic insulin sensitivity was significantly related to SUA. Since skeletal muscles insulin sensitivity is the primary contributor to the whole body insulin sensitivity, it seems reasonable to suppose that the underlying mechanism, which links SUA to 2-h PG, might be the SUA-mediated chronic low-grade inflammation and whole body insulin resistance primarily in skeletal muscles. Moreover, we also found that the association of SUA with 2-h PG persisted even after additional adjustment for CRP and whole body insulin sensitivity (data not shown), indicating the exist of the inflammation- and insulin resistance-independent pathways that links SUA to 2-h PG, which need to be investigated in the future studies.

The strength of the present study includes: the simultaneous measurement of FPG and HbA1c, no usage of any glucose-lowering or SUA-lowering agents, and the population-based design with relatively large sample size and comprehensive adjustment (including HbA1c and FPG). Several limitations of our study need to be considered. First, the cross-sectional design does not allow any causality analysis. Second, selective bias can be introduced, because individuals with relatively lower glucose or HbA1c levels might have a lower participation rate than those with higher glucose or HbA1c levels. Third, some parameters in the present study were estimated by formula or well-accepted methods, such as estimating GFR by the newly CKD-EPI formula and the whole body insulin sensitivity by the OGTT.

In conclusion, SUA in individuals with IFG and/or IA1c is strongly associated with 2-h PG, independent of FPG and HbA1c and other established risk factors, suggesting a significant role of SUA in the deterioration of glucose toleration. Interventional studies are warranted to further investigate the nature of this association.
